# Novel thermostable antibiotic resistance enzymes from the Atlantis II Deep Red Sea brine pool

**DOI:** 10.1111/1751-7915.12468

**Published:** 2016-12-22

**Authors:** Ali H. A. Elbehery, David J. Leak, Rania Siam

**Affiliations:** ^1^ Graduate Program of BiotechnologyThe American University in CairoCairoEgypt; ^2^ Department of Biology and BiochemistryUniversity of BathBathUK; ^3^ Biology Department and YJ‐Science and Technology Research CenterThe American University in CairoCairoEgypt

## Abstract

The advent of metagenomics has greatly facilitated the discovery of enzymes with useful biochemical characteristics for industrial and biomedical applications, from environmental niches. In this study, we used sequence‐based metagenomics to identify two antibiotic resistance enzymes from the secluded, lower convective layer of Atlantis II Deep Red Sea brine pool (68°C, ~2200 m depth and 250‰ salinity). We assembled > 4 000 000 metagenomic reads, producing 43 555 contigs. Open reading frames (ORFs) called from these contigs were aligned to polypeptides from the Comprehensive Antibiotic Resistance Database using BLASTX. Two ORFs were selected for further analysis. The ORFs putatively coded for 3′‐aminoglycoside phosphotransferase [APH(3′)] and a class A beta‐lactamase (ABL). Both genes were cloned, expressed and characterized for activity and thermal stability. Both enzymes were active *in vitro*, while only APH(3′) was active *in vivo*. Interestingly, APH(3′) proved to be thermostable (*T*_m_ = 61.7°C and ~40% residual activity after 30 min of incubation at 65°C). On the other hand, ABL was not as thermostable, with a *T*_m_ = 43.3°C. In conclusion, we have discovered two novel AR enzymes with potential application as thermophilic selection markers.

## Introduction

Red Sea brine pools represent a unique extreme and secluded environment to understand the evolution of biological life (Miller *et al*., 1966). Twenty‐five brine pools have been discovered, to date, along the central rift of the Red Sea (Antunes *et al*., 2011). Atlantis II Deep (ATIID) is the largest and the most intriguing pool because of the multitude of extreme conditions. It has an area of 60 km^2^ and a salinity that is more than seven times that of normal sea water. Due to underlying hydrothermal vent activity, the brine has a temperature of 68°C in addition to high concentrations of different heavy metals (Swift *et al*., 2012). The brine is also anoxic, under relatively high pressure and contains high sulfide concentrations (Siam *et al*., 2012; Swift *et al*., 2012). Salinity and temperature gradients segregate the brine into four layers, the lower convective layer (LCL) and three upper convective layers. LCL is the hottest, saltiest, deepest and most secluded layer of the Atlantis II Deep (Winckler *et al*., 2000). Several studies investigated various functional and phylogenetic aspects of ATIID brine (Abdallah *et al*., 2014; Ferreira *et al*., 2014; Antunes *et al*., 2015; Adel *et al*., 2016). The extreme conditions in LCL stimulated the search for extremophilic organisms and enzymes in this unique environment (Mohamed *et al*., 2013; Sayed *et al*., 2014; Sonbol *et al*., 2016). The properties of these enzymes could explain how indigenous microorganisms have evolved to survive such harsh environmental conditions and could reveal tools for several biotechnological applications.

Antibiotic resistance is a complex problem with substantial health impacts. The Center for Disease Control and Prevention (CDC) reported more than two million antibiotic‐resistant infections per annum in the USA, leading to at least 23 000 deaths (Center for Disease Control and Prevention, 2013). Recently, several studies have revealed antimicrobial resistance genes in diverse environments, not only in clinical settings (Wegley *et al*., 2007; Czekalski *et al*., 2012; Bessa *et al*., 2014). Some of these environments were pristine with no reported human activity or antibiotic contamination (Brown and Balkwill, 2009; Toth *et al*., 2010; Bhullar *et al*., 2012), confirming that antibiotic resistance is ancient, contradicting the notion that it only developed after the discovery of antibiotics (D'Costa *et al*., 2011). Furthermore, these studies complement clinical studies suggesting that environmental microorganisms may act as reservoirs for antimicrobial resistance (Martinez, 2008). Recently, marine environments were specifically depicted as global reservoirs for antimicrobial resistance (Hatosy and Martiny, 2015).

The presumable lack of human impact in Red Sea brine pools qualifies them, as pristine environments, for investigating the presence of antibiotic resistance. In addition, the search for antimicrobial resistance in a high‐temperature environment, such as ATIID, could allow better comprehension of antibiotic resistance in thermophiles and lead to the discovery of novel, thermostable antibiotic resistance genes that would expand the repertoire of antibiotic selective markers used in thermophiles. Therefore, in this study, we used a sequence‐dependent metagenomic approach to unravel two novel antibiotic resistance genes from the lower convective layer of Atlantis II Deep (ATIID‐LCL). Both genes were < 60% identical to already known antibiotic resistance enzymes, at the amino acid level. The genes code for a class A beta‐lactamase (ABL) and an aminoglycoside‐3′‐phosphotransferase APH(3′). Both genes were synthesized, then cloned and overexpressed in *Escherichia coli*. The purified enzymes were assayed for activity and thermostability.

## Results and Discussion

In this study, we used a sequence‐based metagenomic approach to identify two novel antibiotic resistance genes from the lower convective layer of the Atlantis II Deep brine pool (ATIID‐LCL). This deepest part of the ATIID is considered a pristine and poly‐extreme environment (Winckler *et al*., 2000). Antimicrobial resistance genes have been previously identified in marine aquatic environments with no documented anthropogenic impact (Wegley *et al*., 2007; Toth *et al*., 2010). In this context, antimicrobial resistance could be viewed as part of an ongoing attack–defence co‐evolution survival mechanism. Therefore, the study of antibiotic resistance in such environments would allow deeper understanding of the evolution of the antibiotic resistance phenomena. Additionally, the identification of antibiotic resistance enzymes from the hot ATIID‐LCL would be of interest for application as selective marker genes in thermophiles.

### Identification of putative antibiotic resistance genes from the Atlantis II Deep Brine Pool Metagenome data set

#### The Atlantis II brine pool metagenome data set

DNA isolated from the lower convective layer of Atlantis II Deep brine pool (ATIID‐LCL) was shotgun pyrosequenced using Roche‐454. A total of 4 184 386 reads with more than 1.6 billion bp were generated (SRA: SRX1143264). The median read length was 454 bp. The assembly of these reads resulted in 43 555 contigs with a median length of 2371 bp. ORF calling on these contigs gave rise to 89 760 ORFs with a median length of 666 bp.

### Identification of putative Atlantis II antibiotic resistance genes

Translated ORFs were aligned to all polypeptides contained in the Comprehensive Antibiotic Resistance Database (CARD, https://card.mcmaster.ca/) (McArthur *et al*., 2013) with the aim of identifying antibiotic resistance genes. The selection of CARD was performed based on recent recommendations for the identification of antibiotic resistance genes from metagenomics data sets (Elbehery *et al*., 2016; Xavier *et al*., 2016). Indeed, 633 antibiotic resistance ORFs were identified, including multidrug resistance (38%), macrolides (38%), beta‐lactams (7%), tetracyclines (5%), vancomycin (4%), fluoroquinolones (2%) and aminoglycosides (1%). Other less prevalent antibiotic‐resistant ORFs identified include lincosamides, chloramphenicol, rifampin, streptogramin A, bleomycin, polymyxins, aminocoumarins, daptomycin, macrolide, lincosamide and streptogramin B (MLSb phenotype), oxazolidinone and sulfonamides (< 1%). Two ORFs (contig00702_ORF4 and contig00171_ORF16) of ~800–1000 bp were selected for further characterization (Table 1). The criteria that promoted their selection were (i) low per cent identity to known genes, (ii) low e‐values which increased the confidence in their annotation and (iii) they were similar to beta‐lactamases and aminoglycoside kinases, commonly used antibiotic resistance classes in cloning and expression vectors. To confirm the preliminary annotation deduced from BLASTX alignment to CARD, both ORFs were aligned to nr using BLASTX and screened against the conserved domain database (CDD) (Marchler‐Bauer *et al*., 2015) and InterPro (Mitchell *et al*., 2014) Web interfaces. This confirmed that the protein encoded by contig00702_ORF4 belonged to aminoglycoside 3′‐phosphotransferase ATII‐APH(3′), while that encoded by contig00171_ORF16 belonged to class A beta‐lactamase (ATII‐ABL) (Table 1).

**Table 1:** CARD, nr, CDD and Interpro search results for the two ORFs selected for this study

Database	contig00702_ORF4	contig00171_ORF16
CARD BLASTX		
Query Length	804	999
E‐Value	1.00E‐71	1.00E‐26
Description	aph(3p)‐IIa_aac(3)‐II protein [*Escherichia coli*]	extended‐spectrum beta‐lactamase VEB‐4. [*Proteus mirabilis*]
% Identity	53.06	28.71
Hit Coverage	92.42	93.31
nr BLASTX		
E‐Value	1.00E‐100	4.00E‐121
Description	aminoglycoside phosphotransferase [Rhizobium sp. LC145]	beta‐lactamase [Scytonema tolypothrichoides VB‐61278]
% Identity	58	55
Hit Coverage	98.5	98.8
CDD Search		
E‐Value	4.58E‐114	2.23E‐39
Interval	70‐801	1‐993
Accession	cd05150	COG2367
Description	Aminoglycoside 3′‐phosphotransferase (APH).	Beta‐lactamase class A PenP
InterPro		
Protein family membership	Aminoglycoside 3′‐phosphotransferase	Beta‐lactamase, class A
Active Site motif	Not predicted	a(66–81) FSLQSVVKLIVGAAVL

CARD, Comprehensive Antibiotic Resistance Database; nr, NCBI non‐redundant protein database base; CDD, Conserved domain database.

a Amino acid position of the active site.

### Preliminary characterization of the Atlantis II antibiotic resistance genes

**Figure 1:**
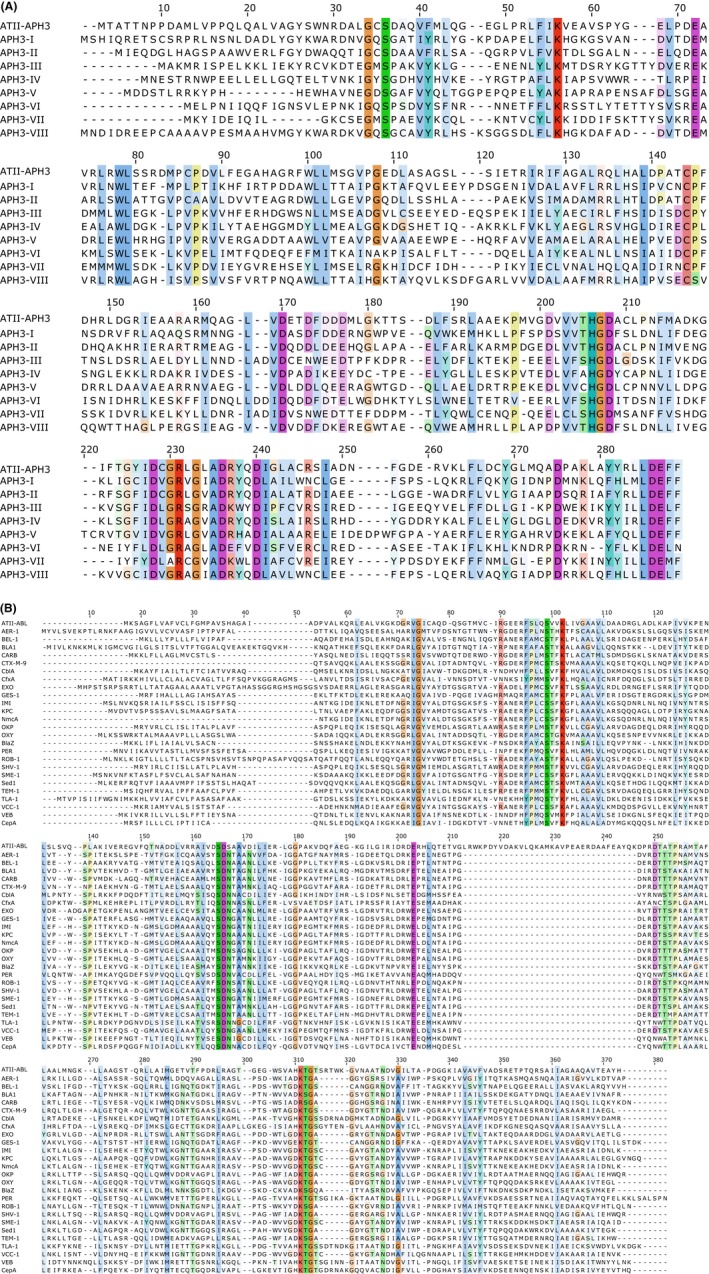
Alignment of ATII‐APH(3′) (A) and ATII‐ABL (B) with representative members of 3′‐aminoglycoside phosphotransferase and class A beta‐lactamase respectively. The alignments were carried out using MUSCLE algorithm in MEGA7. Final images were generated in Jalview v 2.9.0b2 using Clustal X colour scheme, conserved amino acids are shaded, as described‐http://www.jalview.org/help/html/colourSchemes/clustal.html and http://www.jalview.org/help/html/colourSchemes/conservation.html. ATII‐APH(3′) was aligned to eight different 3′‐aminoglycoside phosphotransferases. Accession numbers: APH(3′)‐I, P00551.2; APH(3′)‐II, P00552.1; APH(3′)‐III, P0A3Y6.1; APH(3′)‐IV, P00553.1; APH(3′)‐V, P00555.1; APH(3′)‐VI, P09885.1; APH(3′)‐VII, P14508.1; APH(3′)‐VIII, P14509.1. On the other hand, ATII‐ABL was aligned to 25 different class A beta‐lactamases. Accession numbers: AER‐1, Q44056.2; BEL‐1, 4MXH_A; BLA1, NP_844879.1; CARB, WP_053809595.1; CTX‐M‐9, 1YLJ_A; CblA, WP_005837179.1; CfxA, WP_013618201.1; EXO, WP_033237905.1; GES‐1, 2QPN_A; IMI, WP_050737109.1; KPC, WP_048272923.1; NmcA, 1BUE_A; OKP, WP_060655783.1; OXY, WP_049074725.1; BlaZ, NP_932193.1; PER, WP_001100752.1; ROB‐1, YP_004074575.1; SHV‐1, P0AD64.1; SME‐1, AGZ03855.1; Sed1, AAK63223.1; TEM‐1, YP_006960556.1; TLA‐1, AAD37403.1; VCC‐1, ALU63998.1; VEB, WP_044103626.1; CepA, WP_054958994.1.

**Figure 2:**
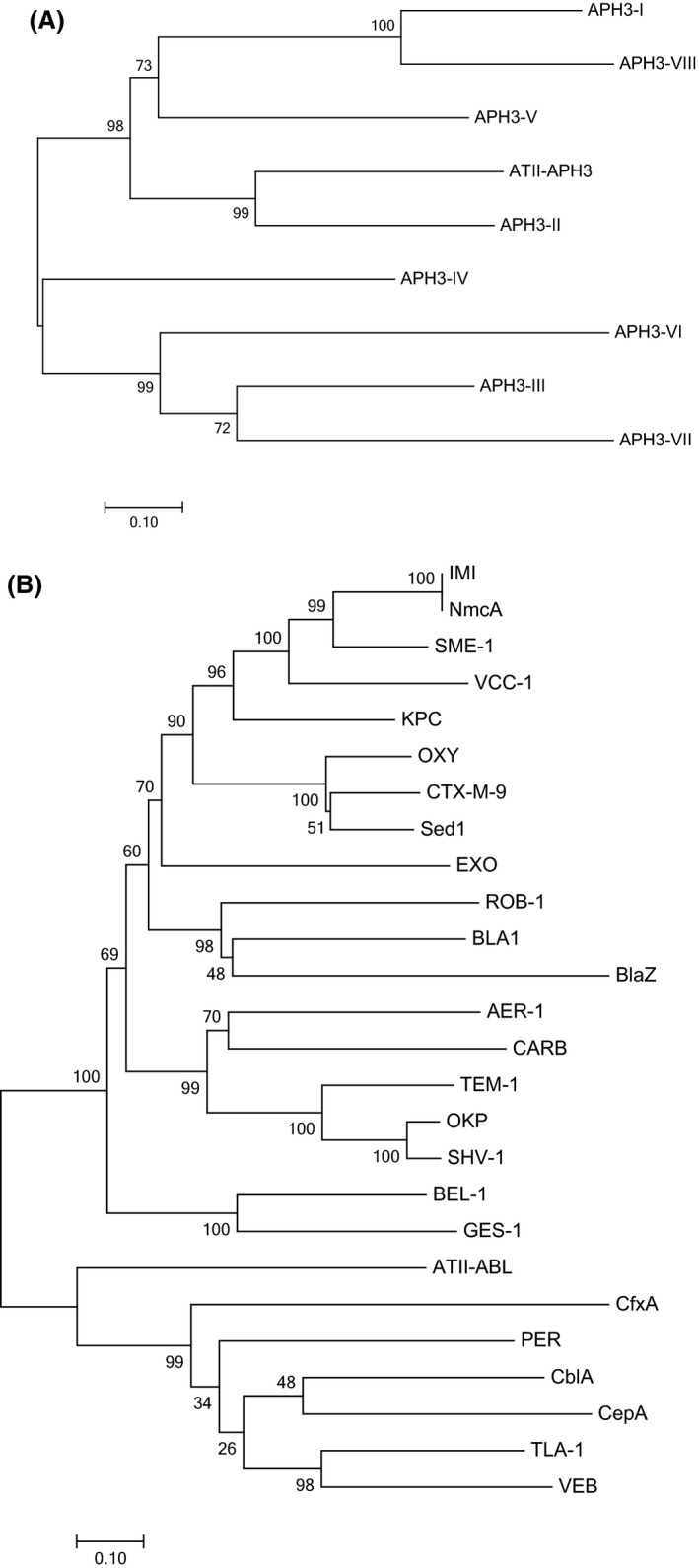
Phylogenetic trees showing (A) ATII‐APH(3′) and (B) ATII‐ABL in relation with representative members of 3′‐aminoglycoside phosphotransferase and class A beta‐lactamase respectively. Trees were generated using Neighbor‐Joining method (Saitou and Nei, 1987) in MEGA7 (Kumar *et al*., 2016). The percentage of replicate trees in which the associated taxa clustered together in the bootstrap test (500 replicates) are shown next to the branches. The tree is drawn to scale, with branch lengths representing the number of amino acid substitutions per site. Accession numbers for 3′‐aminoglycoside phosphotransferases are as follows: APH(3′)‐I, P00551.2; APH(3′)‐II, P00552.1; APH(3′)‐III, P0A3Y6.1; APH(3′)‐IV, P00553.1; APH(3′)‐V, P00555.1; APH(3′)‐VI, P09885.1; APH(3′)‐VII, P14508.1; APH(3′)‐VIII, P14509.1. On the other hand, accession numbers of class A beta‐lactamases are as follows: AER‐1, Q44056.2; BEL‐1, 4MXH_A; BLA1, NP_844879.1; CARB, WP_053809595.1; CTX‐M‐9, 1YLJ_A; CblA, WP_005837179.1; CfxA, WP_013618201.1; EXO, WP_033237905.1; GES‐1, 2QPN_A; IMI, WP_050737109.1; KPC, WP_048272923.1; NmcA, 1BUE_A; OKP, WP_060655783.1; OXY, WP_049074725.1; BlaZ, NP_932193.1; PER, WP_001100752.1; ROB‐1, YP_004074575.1; SHV‐1, P0AD64.1; SME‐1, AGZ03855.1; Sed1, AAK63223.1; TEM‐1, YP_006960556.1; TLA‐1, AAD37403.1; VCC‐1, ALU63998.1; VEB, WP_044103626.1; CepA, WP_054958994.1.

ATII‐APH(3′) was aligned to eight different 3′‐aminoglycoside phosphotransferases, representing major subtypes (Fig. 1A). All residues essential for activity were conserved in ATII‐APH(3′) including Lys52 responsible for ATP binding; Glu65, which orients Lys52 for ATP binding; Asp193, the catalytic residue; and Asn198 and Asp208 responsible for Mg^2+^ binding (Wright and Thompson, 1999). The per cent identity to representative 3′‐aminoglycoside phosphotransferase sequences, ranged from 21.7 to 34.1% in the case of APH(3′)‐VI and APH(3′)‐I respectively. A relatively higher per cent identity (48.7%) was observed with APH(3′)‐II with a high bootstrap value (99%) (Fig. 2A), suggesting that ATII‐APH(3′) belongs to APH(3′)‐II subclass. However, ATII‐APH(3′) showed 36, 20 and 75% higher proline (Pro), serine (Ser) and tyrosine (Tyr) contents, respectively, than APH(3′)‐II (P00552.1). The latter amino acids have been suggested to enhance protein thermal stability (Kumar *et al*., 2000). Additionally, Pro substitutions were detected at amino acid positions: 58, 63, 80 and 254. It was suggested that thermostable proteins use Pro substitutions, in loop areas, to increase protein rigidity and therefore enhance thermal stability (Razvi and Scholtz, 2006). In addition, the relatively higher Ser and Tyr content may enhance protein stability through increasing hydrogen bond interactions (Kumar *et al*., 2000).

Similarly, ATII‐ABL aligned with 25 different class A beta‐lactamases (Fig. 1B), showing the conserved active site motif SXXK corresponding to amino acid positions 70–73, where serine is the catalytic residue. ATII‐ABL also showed a low per cent identity to other class A beta‐lactamases; the lowest was with BlaZ (18.6%), while the highest was with VEB beta‐lactamase (26%). Of note, ABL did not cluster with any of the 25 representative class A beta‐lactamases (Fig. 2B), which could denote a new class A subtype.

### Structure prediction of the proteins encoded by Atlantis II antibiotic resistance genes

**Figure 3:**
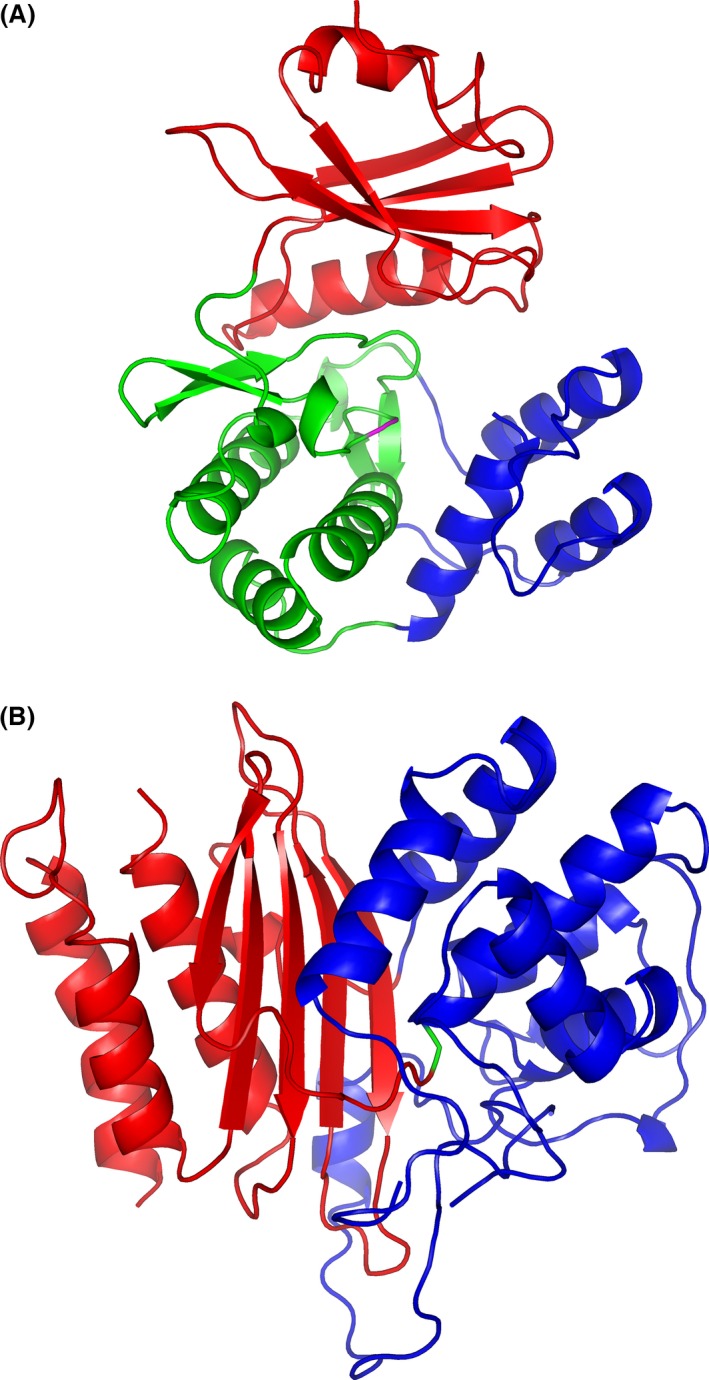
3D‐models for (A) ATII‐APH(3′) and) ATII‐ABL. The structure of APH(3′) is composed of an N‐terminal domain (red) and a C‐terminal domain made of a central core (green) and a helical subdomain (blue). The catalytic residue (Asp193) is shown in magenta. ABL shows two domains: one α‐β domain (red) and another all α‐helical domain (blue). The catalytic residue (Ser70, shown in green) lies in between both domains. Structure prediction was made using PHYRE2 Protein Fold Recognition Server (Kelley *et al*., 2015). Images were generated using pymol v 1.7.2.1.

Structure predictions of the proteins were carried out using the PHYRE2 Protein Fold Recognition Server (Kelley *et al*., 2015). 96% of ATII‐APH(3′) and 84% of ATII‐ABL were modelled with > 90% confidence. The best hit templates (Table S1), used by PHYRE2 server to build up the 3D models, had the same annotations as the query enzymes, confirming the preliminary annotation. The sequence identities of these templates to ATII‐APH(3′) and ATII‐ABL were 52 and 27–37% respectively. 3D‐structure prediction revealed that ATII‐APH(3′) is made up of two domains (Fig. 3A): an N‐terminal domain extending from residues 1–98 and a C‐terminal domain, which is composed of a central core (residues 99–136 & 186–253) and helical subdomain (residues 137–185 & 254–264). The active site lies within the C‐terminal domain. This structure is typical of 3′‐aminoglycoside phosphotransferases, an N‐terminal domain rich in beta‐sheets and a C‐terminal domain rich in alpha‐helices (Nurizzo *et al*., 2003). The crystal structure of APH(3′)‐IIa (PDB ID: 1ND4, Accession:P00552.1) determined by Nurizzo *et al*. (2003) was in fact the template used by PHYRE2 server (Table S1) to predict the 3D structure of ATII‐APH(3′). Interestingly, the two structures superpose (Fig. S1). It is therefore not surprising for ATII‐APH(3′) to have the similar antibiotic binding and activity. Moreover, examining the 3D model of ATII‐APH(3′) showed that the aforementioned Pro substitutions (amino acid positions: 58, 63, 80 and 254) were indeed in the loop areas and helix terminals, suggesting a role in thermal stability, as further discussed below.

On the other hand, ATII‐ABL comprises of two structural domains; α–β domain (residues 1–70 & 254–332) and α‐helical domain (residues 71–253). The catalytic residue (Ser70) lies in between both domains (Fig. 3B). Similarly, ATII‐ABL model exhibited a structure characteristic of class A beta‐lactamases (Joris *et al*., 1991).

The number of salt bridges in ATII‐APH(3′) and ATII‐ABL was examined as a preliminary indicator of thermal stability. It was compared with the respective best hit template from PHYRE2 results. Best hit templates in both cases were from mesophilic organisms (*Klebsiella pneumoniae* and *Pseudomonas aeruginosa*, respectively). However, the number of salt bridges in both Atlantis II enzymes was substantially higher‐ > 7–8 times higher (Table 2). Salt bridges are electrostatic interactions between the ionizable side‐chains (long range) of acidic and basic amino acids (Bosshard *et al*., 2004) and were shown to contribute to thermal stability in proteins (Lam *et al*., 2011; Lee *et al*., 2014). Usually, proteins from thermophiles and hyperthermophiles show overrepresentation of salt bridges compared to their mesophilic homologues, supporting the theory that thermostable proteins benefit from the electrostatic stabilization conferred by salt bridges (Bosshard *et al*., 2004).

**Table 2:** Number of salt bridges in the two novel enzymes (ATII‐APH(3′) and ATII‐ABL) and their corresponding best hit template

Protein	Number of salt bridges	Best hit template PDB ID	Number of salt bridges
ATII‐APH(3′)	146	1ND4	19
ATII‐ABL	116	1E25	14

### Biochemical characterization of the Atlantis II antibiotic resistance genes

#### Protein expression and purification

His‐tagged ATII‐APH(3′) and ATII‐ABL proteins were expressed and purified, as described in ‘Experimental Procedures’. Eluted proteins were more than 95% pure as evident by SDS‐PAGE analysis (Figs S1 and S2). One litre of *E. coli* BL21 (DE3) culture yielded 3.26 mg of ATII‐APH(3′) and 0.147 mg for ATII‐ABL. Purified proteins were tested for enzymatic activity and thermal stability.

#### Enzyme kinetics

The catalytic activity of ATII‐APH(3′) was determined using three thermostable aminoglycoside substrates, namely kanamycin, neomycin and amikacin. ATII‐APH(3′) had *K*_m_ values in the micromolar range (Table 3); 4.7 and 11.3 μm for kanamycin and neomycin respectively. In contrast, the *K*_m_ for amikacin was ~1000‐fold higher (5.5 mm). *K*_m_ values were therefore lowest for kanamycin and highest for amikacin. The difference in *K*_m_ values indicates that ATII‐APH(3′) has a much lower affinity for amikacin compared to kanamycin, despite their similarity in structures. This difference in affinity must reflect the presence of the (S)‐4‐amino‐2‐hydroxybutyryl substitution at the N1 of the 2‐deoxystreptamine ring in amikacin (Fig. S2). This group is believed to impede binding to 3′‐aminoglycoside phosphotransferase (McKay *et al*., 1994; Mingeot‐Leclercq *et al*., 1999). On the other hand, the turnover number (*k*_cat_) was highest with neomycin followed by amikacin, then kanamycin. Overall, the catalytic efficiency (*k*_cat_/*K*_m_) of ATII‐APH(3′) was highest with neomycin (1.996 s^−1^ μm ^−1^), three times lower for kanamycin and the lowest for amikacin (> 1600 times lower than neomycin). Similar kinetic parameters were previously reported for 3′‐ aminoglycoside phosphotransferase type II (McKay *et al*., 1994) and type III (Hainrichson *et al*., 2007) with one exception‐*k*_cat_/*K*_m_ was higher for kanamycin compared to neomycin.

**Table 3:** Enzyme kinetic parameters *K*_m,_ *k*_cat_ and catalytic efficiency *k*_cat_/*K*_m_ for ATII‐APH(3′) and ATII‐ABL

Enzyme	Antibiotic	*K*_m_	*k*_cat_ (s^−1^)	*k*_cat_/*K*_m_ (s^−1^ μm ^−1^)
ATII‐APH(3′)	Kanamycin	4.7 ± 0.96 μm	3.2 ± 0.22	0.68
	Neomycin	11.3 ± 1.89 μm	22.55 ± 0.8	1.996
	Amikacin	5.5 ± 1.65 mm	6.4 ± 0.78	0.0012
ATII‐ABL	Nitrocefin	5.065 ± 1.65 μm	0.91 ± 0.07	0.18

**Figure 4:**
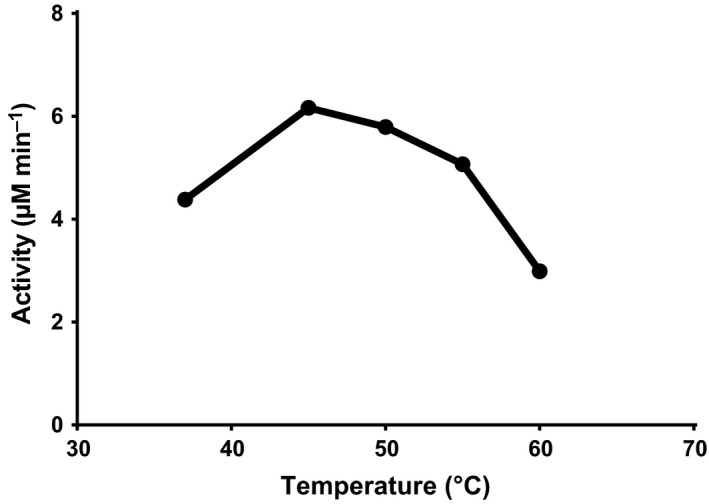
Variation of ATII‐ABL enzyme activity with temperature. ABL enzyme activity was determined using nitrocefin at 37, 40, 45, 50, 55 and 60°C. The initial reaction velocity was monitored for 1 min.

Measurement of the kinetic parameters of ATII‐ABL showed a *K*_m_ in the micromolar level with nitrocefin (Table 3), turnover number of 0.91 s^−1^ and resulting catalytic efficiency of 0.18 s^−1^ μm ^−1^_._ The effect of temperature on the enzyme activity was investigated (Fig. 4), and 45°C was the optimum temperature for enzyme activity. This activity profile of ATII‐ABL is different from other class A beta‐lactamases as KPC‐1 (Yigit *et al*., 2001) and TEM‐1 (Bebrone *et al*., 2001). While the *K*_m_ for nitrocefin was 4.5 and 10 times lower than that of KPC‐1 and TEM‐1, respectively, the *k*_cat_ was 85 and > 1000 times lower leading to very low catalytic efficiency.

### A kanamycin and neomycin antibiotic resistance gene from the Atlantis II brine pool

Minimum inhibitory concentration (MIC) determination experiments were conducted using *E. coli* BL21 (DE3) transformed with pET vectors containing the genes of interest. As the main aim of our study was to identify antibiotic‐resistant enzyme that may be used as selection markers in thermophiles, our MIC experiment utilized commonly used aminoglycosides with documented thermal stability (Connors *et al*., 1986; Traub and Leonhard, 1995). Expression of ATII‐APH(3′) resulted in both kanamycin and neomycin resistance, and the MIC levels increased > 32‐fold and eightfold, respectively, compared to the control (Table 4). In contrast, the MIC remained the same as the control, in case of amikacin. Previous studies of APH(3′)‐II showed similar results, where increased tolerance of transformed expression hosts to kanamycin was observed in comparison with neomycin, and was negligible for amikacin (Nurizzo *et al*., 2003; Hainrichson *et al*., 2007). This demonstrates that tolerance is primarily a reflection of *K*_m_ value rather than *k*_cat_/*K*_m_. In contrast, ATII‐ABL did not confer resistance to the beta‐lactam antibiotics tested, as no increase in control baseline MIC levels was observed when this was expressed. It is likely that the low activity of the enzyme combined with the low expression level (147 μg l^−1^) in *E. coli* BL21 (DE3) may have contributed to the lack of resistance in our MIC assay, compared to the non‐transformed *E. coli* BL21 (DE3) cells.

**Table 4:** Results of minimum inhibitory concentration (MIC) experiments

	Antibiotic	MIC (μg ml^−1^)	MIC for control^a^ (μg ml^−1^)
ATII‐APH(3′)	Kanamycin	> 512	16
	Neomycin	128	16
	Amikacin	16	16
ATII‐ABL	Ampicillin	2	2
	Oxacillin	8	8
	Azlocillin	8	8

a Non‐transformed *Escherichia coli* BL21 (DE3).

### A thermally stable Atlantis II antibiotic resistance gene

**Figure 5:**
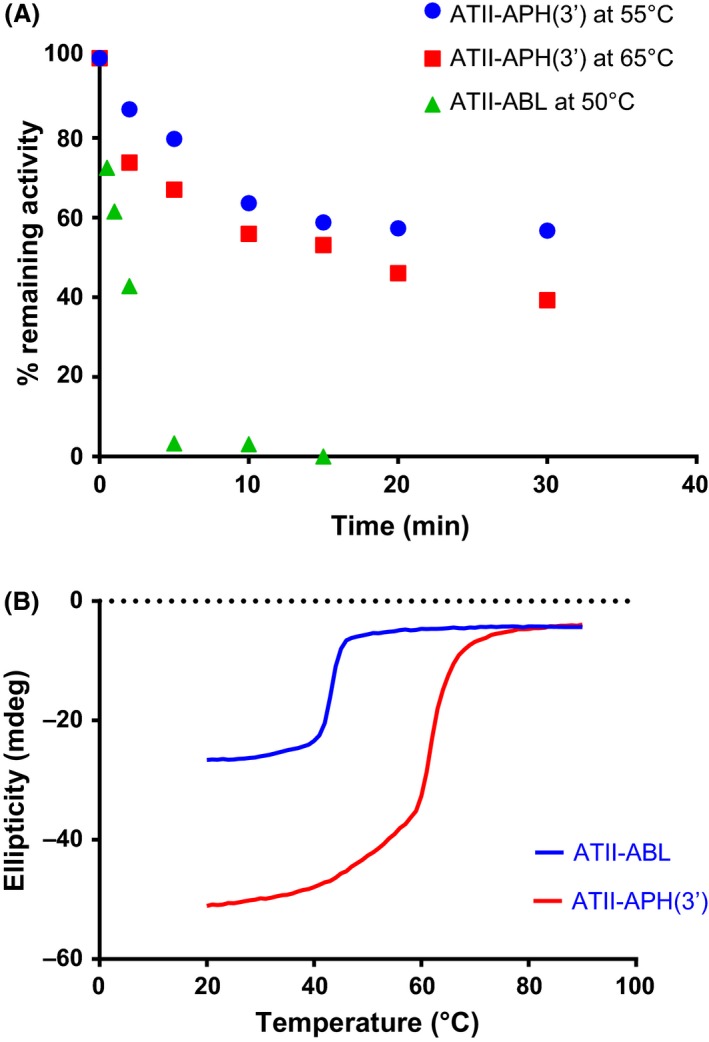
Thermal stability of ATII‐APH(3′) and ATII‐ABL. (A) Scatter plot showing % remaining activity for both enzymes after incubation for increasing amounts of time at elevated temperatures. (B) Circular dichroism melting curves showing the change in ellipticity with temperature increase from 20 to 90°C at 222 nm.

Thermal stability was tested by evaluating enzyme activity following incubation at high temperatures and also by investigating loss of secondary structure, using circular dichroism. The enzymatic activity was recorded at different temperatures and durations. This approach showed that ATII‐APH(3′) possesses appreciable thermal stability, with ~40% of the enzyme activity retained following 30 min incubation at 65°C (Fig. 5A). On the other hand, this method could not detect any significant thermostability with ATII‐ABL; the enzymatic activity was remarkably reduced following incubation at 50°C. Less than 50% activity were retained following a 2 min incubation, and activity was lost after 5 min (Fig. 5A).

Both enzymes were scanned in a CD spectrometer between 200 and 300 nm, and both showed maximal ellipticity at 208 nm (Fig. S3), indicating high helical content (Greenfield, 2006). This finding allowed monitoring of protein unfolding at 222 nm during a temperature ramp from 20 to 90°C (Fig. 5B). Second‐derivative plots of the melting curves (Fig. S4) showed that the melting temperatures (*T*_m_) for ATII‐APH(3′) and ATII‐ABL were 61.7 and 43.3°C respectively.

ATII‐APH(3′) is the first example of a naturally thermostable 3’‐aminoglycoside phosphotransferase; no other thermostable example of this class has been previously reported. The only other reported example was from a different class; 4‐aminoglycoside phosphotransferase‐Ia (APH(4)‐Ia) (also known as hygromycin B phosphotransferase) (Nakamura *et al*., 2005). The latter enzyme was thermostabilized using *in vivo‐*directed evolution and was successfully used to grow *Thermus thermophilus* in the presence of hygromycin at 67°C. *T*_m_ was determined to be 58.8°C (Nakamura *et al*., 2008). However, this enzyme is only active against hygromycin, the only aminoglycoside with a free 4‐hydroxyl group. ATII‐APH(3′), in contrast, is active against both kanamycin and neomycin, while its *T*_m_ is slightly higher (61.7°C). Given that it is naturally thermostable, it may be feasible to increase the thermal stability via directed evolution. Of note, few other thermally stable aminoglycoside modifying enzymes, belonging to the nucleotidyltransferase group, can mediate resistance to kanamycin (Matsumura *et al*., 1984, Liao *et al*., 1986, Hoseki *et al*., 1999).

Despite the evidence for a higher number of salt bridges compared to its mesophilic equivalents, ABL was not as thermostable as APH(3′). It showed rapid inactivation after incubation at 50°C, which could be understood in view of its *T*_m_ determined by CD, which was 43.3°C. Optimal thermoactivity of the enzyme was observed at 45°C, in agreement with the determined *T*_m_. It is worth noting that an increase in temperature over 45°C leads to a simultaneous increase in the enzyme inactivation rate as well as the catalytic rate. Therefore, enzyme activity above 45°C starts to decrease leading to a bell‐shaped curve (Fig. 4). Although the *T*_m_ of the enzyme is relatively low for use in thermophilic hosts, it could still be of interest for moderate thermophiles and/or thermotolerant organisms, particularly as some enzymes *in vivo* can withstand temperatures higher than their *T*_m_, as was the case with APH(4)‐Ia (Nakamura *et al*., 2005). Generally, beta‐lactamase resistance in thermophiles is poorly characterized. Only one study has described a thermostable beta‐lactamase, which was isolated from a thermophilic *Bacillus* from a Moroccan hot public bath (Rhazi‐Filali *et al*., 1996). Unfortunately, the authors did not sequence the gene encoding this enzyme.

In conclusion, we have identified and characterized two novel antibiotic resistance enzymes from the Atlantis II Red Sea brine pools and report the first thermostable 3’‐aminoglycoside phosphotransferase. Further work may shed light on two important and poorly studied issues including (i) evolution of antibiotic resistance in thermophilic environments and (ii) role of antibiotic resistance in extreme and pristine sites as defence tools in a continuously ongoing survival battle. Furthermore, these antibiotic resistance genes can potentially be used as selective marker genes in thermophilic hosts, enriching the thermophilic selection marker gene repertoire.

## Experimental procedures

### Sample collection, DNA extraction and sequencing

In April 2010 on board of the research vessel *Aegaeo*, second leg of KAUST/WHOI/HCMR Red Sea expedition, water samples from ATIID‐LCL were collected as previously described (Abdallah *et al*., 2014). Water underwent sequential filtering steps using 3.0, 0.8 and 0.1 μm filters. DNA was extracted from the fraction retained on the 0.1 μm filter as previously described (Fàbrega *et al*., 2009) and sequenced using a GS FLX pyrosequencer with the Titanium pyrosequencing kit (454 Life Sciences) after preparing the DNA libraries according to manufacturer's instructions. Metagenomic reads were quality controlled using PRINSEQ‐lite v0.20.4 (Schmieder and Edwards, 2011) and CD‐HIT‐454 (Niu *et al*., 2010).

### Contig assembly and bioinformatic analysis

Contigs were assembled using the GS assembler (The GS Data Analysis Software package, 454 Life Sciences) with default parameters. Assembly was followed by open reading frame (ORF) calling using Artemis (Rutherford *et al*., 2000). ORFs were aligned versus all polypeptides contained in the Comprehensive Antibiotic Resistance Database (CARD, https://card.mcmaster.ca/) (McArthur *et al*., 2013) using BLASTX (Altschul *et al*., 1990). The E‐value was set to < 1e‐5, while hit coverage was at least 90%. ORFs of interest were further aligned against the National Center for Biotechnology Institute (NCBI) non‐redundant protein database (nr) using BLASTX. In addition, the annotation of these ORFs was confirmed using both the NCBI's conserved domain database (CDD) (Marchler‐Bauer *et al*., 2015) and InterPro (Mitchell *et al*., 2014). Multiple sequence alignments of proteins were performed using MUSCLE algorithm (Edgar, 2004), while phylogenetic trees were inferred using the Neighbor‐Joining method (Saitou and Nei, 1987) with bootstrap (Felsenstein, 1985) testing, using 500 replicates. Alignments and trees were generated in MEGA7 (Kumar *et al*., 2016). Viewing and colour editing of alignments were performed with Jalview (Waterhouse *et al*., 2009). We performed 3D‐modelling of the proteins using the PHYRE2 Protein Fold Recognition Server (Kelley *et al*., 2015). Predicted atomic coordinates were used to predict the number of salt bridge in each protein using ESBRI (Evaluating the Salt BRIdges in Proteins) (Costantini *et al*., 2008) with default parameters. The number of salt bridges was similarly predicted in the corresponding best hit template from PHYRE2 results. If the Protein Data Bank (PDB) file for the best hit template contained more than one chain (e.g. the protein was homodimer), only one chain was used in the estimation of salt bridges.

### Gene synthesis, cloning and transformation

The retrieved sequence of the APH(3′)‐encoding gene was modified to include NdeI and BamHI restriction sites to allow in‐frame cloning into pET‐16b (Novagen, Madison, WI, USA) with an N‐terminal 10x‐His tag. The sequence was codon‐optimized for expression in *E. coli* using the GeneArt™ Web interface and the gene synthesized by GeneArt™ (Thermo Fisher Scientific, Waltham, MA, USA).

The class A beta‐lactamase gene encoded a signal sequence, as identified using the SignalP 4.1 server (Nielsen *et al*., 1997). The native signal sequence was replaced with a pelB leader (Lei *et al*., 1987) and the resulting sequence modified to include NcoI and XhoI restriction sites to allow in‐frame cloning into pET‐28a(+) with a C‐terminal 6x‐His tag. The sequence was similarly codon‐optimized for expression in *E. coli* and synthesized by GeneArt^™^.

Genes were released from the supplier's holding vectors using either NdeI and BamHI [for APH(3′)] or NcoI and XhoI (for ABL) restriction enzymes (FastDigest; Thermo Fisher Scientific), then gel purified using Zymoclean™ Gel DNA Recovery Kit (Zymo Research, Irvine, CA, USA). The fragments were then ligated with their target pET vectors, similarly restriction digested, using T4 ligase (Thermo Fisher Scientific). The 20 μl reaction contained 3:1 gene‐to‐vector molar ratio in addition to 2 U of T4 ligase and was incubated at room temperature for 1 h. Two microlitres of the mixture was then transformed into BIOBlue chemically competent *E. coli* (Bioline, London, UK) using heat shock (Froger and Hall, 2007), and positive clones were identified by colony PCR using T7‐promoter and terminator primers. The PCR was performed using REDTaq^®^ ReadyMix™ (Sigma) in a GenePro thermal cycler (Bioer Technology, Binjiang, Zhejiang, China); denaturation at 95°C for 3 min, 35 cycles of denaturation at 95°C for 30 s, annealing at 43°C for 30 s and extension at 72°C for 1 min 15 s; final extension was performed at 72°C for 5 min. Recombinant plasmids were extracted from positive clones and inserts sequenced, in both directions, using T7 primers. Sequencing was performed by GATC BIOTECH (Konstanz, Germany). Plasmid constructs were extracted using QIAprep Spin Miniprep Kits (Qiagen, Venlo, Netherlands) according to the manufacturer's instructions, then transformed into chemically competent *E. coli* BL21 (DE3) (Novagen) for protein expression.

### Protein expression and purification

#### APH(3′)

An overnight culture of *E. coli* BL21 (DE3) transformed with pET‐16b containing the APH(3′) gene was grown on lysogeny broth (LB, a litre of medium contains 10 g tryptone (Melford Laboratories Ltd., Ipswich, UK), 5 g yeast extract (Melford Laboratories Ltd.) and 10 g NaCl) containing 100 μg ml^−1^ of ampicillin, in a shaking incubator at 37°C and 200 rpm. Twenty ml of this culture was used to inoculate 1 l of LB containing 100 μg ml^−1^ of ampicillin. The culture was grown at 37°C to an optical density at 600 nm (OD_600_) of ~0.5, when 0.1 mm of the inducer, Isopropyl β‐D‐1‐thiogalactopyranoside (IPTG) was added, followed by a further incubation at 37°C for 5 h. A cell pellet was collected by centrifugation (1500 × g for 15 min) and re‐suspended at 2.5 ml gm^−1^ cells in His‐binding buffer [20 mm Tris pH 8.0, 300 mm NaCl and 10 mm imidazole (Acros Organics; Thermo Fisher Scientific)]. The cell suspension was sonicated on ice for three 30 s bursts each separated by 30 s pauses, using a Soniprep 150 Plus (MSE, London, UK). Cell lysate was collected after centrifugation at 15600 × g for 10 min and applied to a Talon metal affinity resin (Clontech, Mountain View, CA, USA). After washing the resin according to the manufacturer's instructions, APH(3′) was eluted using His‐elution buffer (20 mm Tris pH 8.0, 300 mm NaCl and 200 mm imidazole). APH(3′) protein was checked for purity by running on SDS‐PAGE (Fig. S5), while its concentration was determined using Bradford assay (Bio‐Rad protein assay; Bio‐Rad, Hercules, CA, USA). The protein was preserved in aliquots at −80°C after addition of glycerol to a final concentration of 10%.

#### ABL

An overnight culture of *E. coli* BL21 (DE3) transformed with pET‐28a(+) containing the ABL gene was grown on LB containing 30 μg ml^−1^ kanamycin in a shaking incubator at 37°C and 200 rpm. A total of five litres of LB containing 30 μg ml^−1^ of kanamycin were inoculated with the overnight culture (20 ml of culture per litre of medium). The culture was incubated at 37°C until the OD_600_ reached ~0.5 followed by induction, by adding 0.1 mm IPTG, and incubated at 18°C for 16 h. A cell pellet was collected by centrifugation (as above) and the periplasmic fraction was obtained using a slightly modified osmotic shock method (Neu and Heppel, 1965), in which spheroblasts were gently shaken with ice‐cold Milli‐Q water at 2.5 instead of 80 ml per gm. The supernatant, containing the periplasmic fraction, was collected and dialysed against His‐binding buffer for 2 h at 4°C to enable purification of ABL using Talon metal affinity resin as described for APH(3′). Protein purity was checked by running on SDS‐PAGE (Fig. S6).

### Enzyme assay

#### APH(3′)

Enzyme activity was determined using a coupled assay in which pyruvate kinase and lactate dehydrogenase were used to measure the phosphorylation of the aminoglycoside antibiotic, through determining the rate of oxidation of NADH. The reaction was carried out as described in Kramer and Matsumura (2013) with few modifications in the concentration of some reagents: 0.125 mg ml^−1^ NADH, 4 U ml^−1^ pyruvate kinase and 3.5 U ml^−1^ lactate dehydrogenase. The reaction was monitored for 2 min by following the reduction in NADH absorbance at 340 nm using Cary 50 Bio UV‐Visible Spectrophotometer (Varian, Palo Alto, CA, USA). The initial velocities of APH(3′) obtained at different aminoglycoside concentrations were used to determine the steady state constants *K*_*m*_ and *k*_cat_ by nonlinear regression curve fitting to the Michaelis–Menten equation [graphpad prism version 6.01 for Windows (GraphPad Software, La Jolla, CA, USA, www.graphpad.com)].

#### ABL

ABL activity was assayed using the chromogenic substrate nitrocefin (Toku‐E, Bellingham, WA, USA) as previously described (O'Callaghan *et al*., 1972) using 100 nM of ABL. Colour production was monitored for 1 min at 482 nm, and ABL initial velocity was calculated using a nitrocefin molar extinction coefficient (ε_482_) of 15 900 m ^−1^ cm^−1^. Similar to APH(3′), *K*_m_ and *k*_cat_ were calculated using graphpad prism v. 6.01. Thermoactivity of the enzyme was assessed by monitoring the initial rates of the reaction at 37, 45, 50, 55 and 60°C for 1 min using 100 μm of nitrocefin and 100 nM of ABL. Nitrocefin was not hydrolysed spontaneously (in the absence of enzyme) at the elevated temperatures over the 1 min time span.

### Minimum inhibitory concentration (MIC) experiments

MIC experiments were performed using the macrodilution method as described by the Clinical Laboratory Standards Institute (CLSI) (CLSI, 2012). Briefly, a standard inoculum of the bacteria under investigation was prepared by adjusting the turbidity of the bacterial suspension to an OD_600_ between 0.125 and 0.25, which is equivalent to the turbidity of the 0.5 McFarland standard and a cell density of 1–2 × 10^8^ CFU ml^−1^. Then, 1:150 dilution of the inoculum was prepared and 1 ml of this dilution was added to each tube of the twofold antibiotic dilution series, over the concentration range 0.125–512 μg ml^−1^. The tubes were incubated at 37°C for 24 h and MIC determined as the lowest antibiotic concentration showing no turbidity. *E. coli* BL21 (DE3) expressing APH(3′) was tested against kanamycin, neomycin and amikacin, and *E. coli* BL21 (DE3) expressing ABL against ampicillin, oxacillin and azlocillin.

### Thermostability

Aliquots of 50 μl of the enzyme in microfuge tubes were incubated for varying periods of time at specific temperatures. The tubes were centrifuged 15600 × g for 10 min to spin down any precipitated enzyme. The supernatant was assayed for activity as described above, and per cent remaining activity was calculated relative to enzyme activity with no thermal treatment.

Enzyme melting curves were also determined using far UV circular dichroism (CD) to serve as another measure of thermal stability. The buffer used for APH(3′) consisted of 20 mm Tris pH 7.6 and 100 mm potassium fluoride, while for ABL, it was 20 mm potassium phosphate pH 7.0 and 100 mm potassium fluoride. The concentrations of APH(3′) and ABL were 12 and 11 μm respectively. Each enzyme was placed in a rectangular cuvette of 1 mm path length (Hellma Analytics, Müllheim, Germany). Enzymes were first scanned using a Chirascan CD Spectrometer (Applied Photophysics, Leatherhead, UK) between 200 and 300 nm while recording every 1 nm for 0.5 s per nm with a bandwidth of 1 nm. The scan was the average of three repeats for each wavelength. Then, melting curves for the enzymes were obtained by monitoring the CD at 222 nm over a temperature ramp from 20 to 90°C. The ramp rate was of 1°C per min in steps of 1 °C. At each temperature, the enzyme was allowed to equilibrate for 30 s before recording the CD. The tolerance was 0.1 °C, and data were taken for 5 s per degree. Melting temperatures (*T*_m_) were obtained from the second‐derivative plots of the melting curves.

### Accession numbers

GenBank accessions for genes encoding APH(3′) are KX377799 (natural sequence) and KX377800 (codon‐optimized sequence), while accessions for genes encoding ABL are KX377801 (natural) and KX377802 (codon‐optimized).

Metagenomic sequences are available through NCBI's Sequence Read Archive (SRA), accession number: SRX1143264.

## Conflict of interest

Authors declare no conflict of interest.

## Supporting information


**Fig. S1.** Superposition of ATII‐APH(3′) and APH(3′)‐IIa (PDB ID: 1ND4). ATII‐APH(3′) is shown in green, while APH(3′)‐IIa is shown in cyan.
**Fig. S2.** Chemical structures of amikacin and kanamycin.
**Fig. S3.** Circular dichroism scans for ATII‐APH(3′) and ATII‐ABL between 200 and 300 nm.
**Fig. S4.** Second‐derivative plots for melting curves of (A) ATII‐APH(3′) and (B) ATII‐ABL.
**Fig. S5.** SDS‐PAGE analysis of purified ATII‐APH(3′).
**Fig. S6.** SDS‐PAGE analysis of purified ATII‐ABL.
**Table S1.** Best hit templates used by the PHYRE2 server to build 3D structure models for ATII‐APH(3′) and ATII‐ABL.
