# Supplementary material for: Novel thermostable antibiotic resistance enzymes from the Atlantis II Deep Red Sea brine pool
Source: Microb Biotechnol. 2016 Dec 22;10(1):189–202. doi: 10.1111/1751-7915.12468 (PMC5270753; doi:10.1111/1751-7915.12468)
Supplement: Supplementary file 1 — Fig. S1. Superposition of ATII‐APH(3′) and APH(3′)‐IIa (PDB ID: 1ND4). ATII‐APH(3′) is shown in green, while APH(3′)‐IIa is shown in cyan. Fig. S2. Chemical structures of amikacin and kanamycin. Fig. S3. Circular dichroism scans for ATII‐APH(3′) and ATII‐ABL between 200 and 300 nm. Fig. S4. Second‐derivative plots for melting curves of (A) ATII‐APH(3′) and (B) ATII‐ABL. Fig. S5. SDS‐PAGE analysis of purified ATII‐APH(3′). Fig. S6. SDS‐PAGE analysis of purified ATII‐ABL. Table S1. Best hit templates used by the PHYRE2 server to build 3D structure models for ATII‐APH(3′) and ATII‐ABL. [file MBT2-10-189-s001.docx]

**
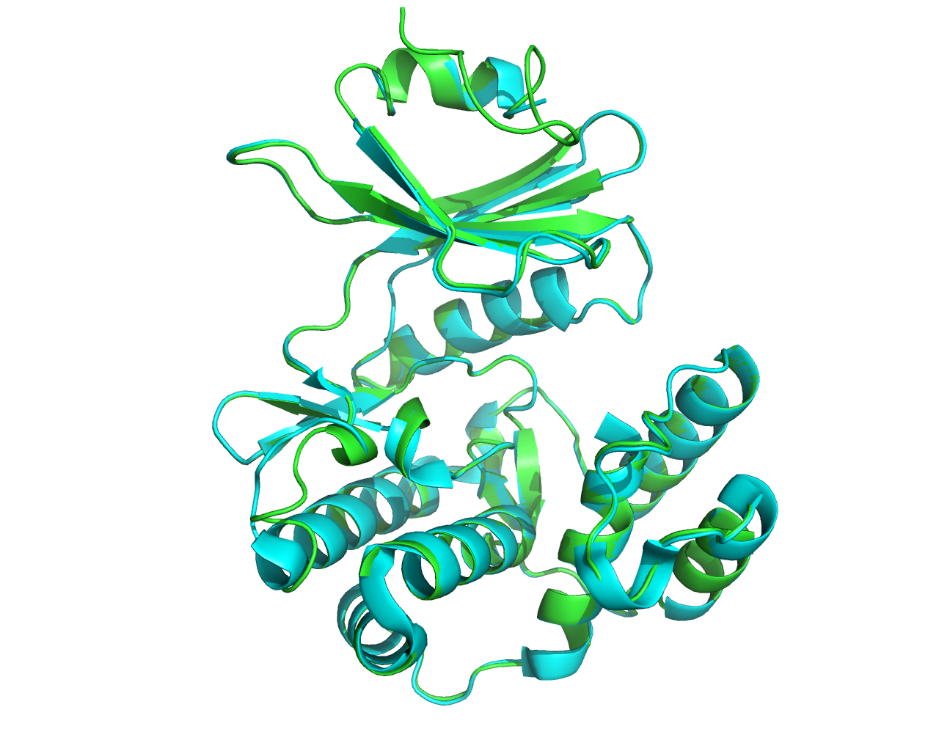
**

**Figure S1. Superposition of ATII-APH(3') and APH(3')-IIa (PDB ID: 1ND4).** ATII-APH(3') is shown in green, while APH(3')-IIa is shown in cyan. Structural alignment and figure preparation were done using PyMOL v1.7.2.1.

Amikacin

Kanamycin

**S2.**

**Circular dichroism scans for ATII-APH(3') and ATII-ABL between 200 and 300 nm**

**Second derivative plots for melting curves of A) ATII-APH(3') and B) ATII-ABL**


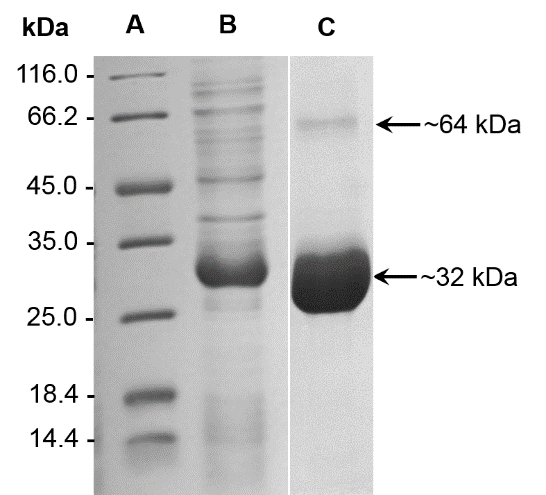


**SDS-PAGE analysis of purified ATII-APH(3').** Lane A, Pierce™ Unstained Protein MW Marker (ThermoFisher Scientific), size in kDa is shown on the left; Lane B, total soluble fraction; Lane C, purified ATII-APH(3'). A faint band of double the size of the ATII-APH(3') monomeris visible in lane C, which could be incompletely reduced ATII-APH(3') dimer. Gels spliced for labeling purposes.


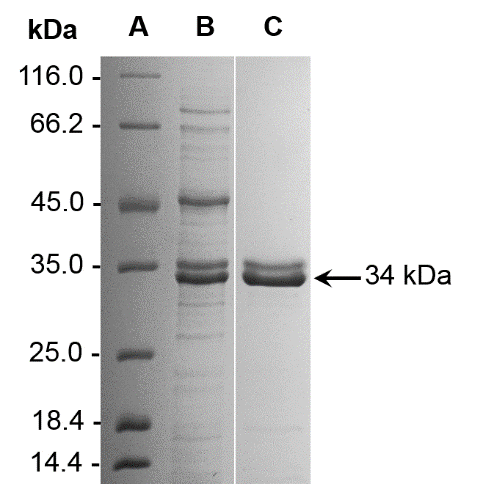


**Figure S6. SDS-PAGE analysis of purified ATII-ABL.** Lane A, Pierce™ Unstained Protein MW Marker (ThermoFisher Scientific), size in kDa is shown on the left; Lane B, periplasmic extract; Lane C, purified ATII-ABL. A higher molecular weight band which co-purifies with ATII-ABL presumably results from incomplete processing of the signal peptide. Gels spliced for labeling purposes.

**Table S1. Best hit templates used by the PHYRE2 server to build 3D structure models for ATII-APH(3') and ATII-ABL**

| Protein | Best hit template PDB ID | % identity | Description |
| --- | --- | --- | --- |
| ATII-APH(3') | 1ND4 | 52 | Aminoglycoside-3'-phosphotransferase-IIa, *Klebsiella pneumoniae* |
| ATII-ABL | 1E25 | 31 | PER-1 class A beta-lactamase, *Pseudomonas aeruginosa* |
|  | 1G6A | 27 | PSE-4 carbenicillinase, *Pseudomonas aeruginosa* |
|  | 1N9B | 32 | SHV-2 enzyme, a class A beta-lactamase, *Klebsiella pneumoniae* |
|  | 4B88 | 37 | Ancestral (GNCA) Beta-lactamase class A |
|  | 4EQI | 28 | SFC-1 carbapenemase, *Serratia fonticola* |
|  | 2OV5 | 30 | KPC-2 carbapenemase, *Klebsiella pneumoniae* |
